# Utilization of public health services and health outcomes among China's migrant population: an equity analysis

**DOI:** 10.3389/frhs.2026.1708382

**Published:** 2026-04-29

**Authors:** Bo Dong, Qianqian Hu, Yuxin Zhou, Shanshan Cai, Yachao Li, Xuehong Zhang

**Affiliations:** 1 Department of Urology, Nanchang County People's Hospital, Nanchang, Jiangxi, China; 2 School of Public Health, Zhejiang Chinese Medicine University, Hangzhou, Zhejiang, China; 3 Linyi People's Hospital, Linyi, Shandong, China; 4 School of Marxism, Xi'an Jiaotong University, Xi'an, Shaanxi, China; 5 Division of Biomedical and Life Sciences, Faculty of Health and Medicine, Lancaster University, Lancaster, United Kingdom; 6 School of Computer and Electronic Information Engineering, Wuhan City Polytechnic, Wuhan, Hubei, China

**Keywords:** basic public health service utilization, China, equity, health, migrant population

## Abstract

**Background:**

The equalization of basic public health services is a core component of China's healthcare reform, which is aimed at promoting the health of the general population. Improving migrants’ access to these services and enhancing their health outcomes are important objectives of this policy. While previous studies have documented income-related inequalities in the utilization of public health services and health status among migrant populations, insufficient attention has been paid to the impact of public health service utilization on health outcomes and the realization of health equity.

**Purpose:**

This study examined the relationship between public health service utilization and health outcomes among China's migrant population. Specifically, it assessed equity in public health service utilization and health status, analyzed the effects of service utilization on health outcomes, and explored the implications of these services for health equity.

**Methods:**

Data were drawn from the 2018 China Migrants Dynamic Survey, yielding a final sample of 4,034 participants after data cleaning. The corrected concentration index was used to assess equity in the utilization of four public health services—health education, health record establishment, family doctor contracting, and inpatient care—as well as equity in health status. Ordered logistic regression models were applied to estimate the effects of public health service utilization on health outcomes. In addition, concentration index decomposition was conducted to evaluate the contribution of service utilization to health equity and to examine heterogeneity across subgroups.

**Results:**

Significant disparities were observed in public health service utilization among migrants. Health education had relatively high coverage, whereas health record establishment, family doctor contracting, and inpatient care were underutilized. Income-related inequalities were evident in service utilization: health education showed a pro-rich distribution, while health record establishment, family doctor contracting, and inpatient care showed pro-poor distributions. Although migrants generally reported good self-rated health, notable income-related health inequalities persisted. Overall, all four types of public health services were associated with better health status, with preventive services showing stronger effects than inpatient care. In terms of health equity, health education tended to widen health inequalities, whereas health record establishment and family doctor contracting helped reduce them. Heterogeneity analysis further indicated that these effects were more pronounced among socioeconomically disadvantaged migrant subgroups.

**Conclusion:**

Public health service utilization and its equity vary substantially across different service types among China's migrant population. Although these services generally contribute to improved health status, their impacts on health equity differ significantly. Policies should therefore focus on expanding access to essential public health services and tailoring service delivery to the specific needs of disadvantaged migrant groups, with the aim of enhancing both health outcomes and health equity.

## Introduction

In China, the term “migrant population” refers to individuals whose current place of residence differs from their registered residence (hukou), commonly known as “non-local residents.” (1). This group has served as a vital driving force behind China's rapid economic development (2, 3), with migration often representing a crucial survival strategy—particularly for the vast number of people living in rural areas (4). China hosts the world's largest migrant population (5). According to data from the 2021 Seventh National Population Census, the migrant population reached nearly 380 million in 2020—an increase of 150 million compared to 2010, representing nearly 70% growth over a decade (6). The migration of this population not only injects vitality into local economies but also provides them with broader development opportunities (7, 8). Despite significant economic gains, migrant workers still lag far behind local residents in access to public health and social security. Most remain marginalized, without equal access to the social support and services available to residents.

Health inequalities among migrant populations primarily stem from health-related social determinants such as socioeconomic status, educational attainment, and access to information. Due to their generally lower socioeconomic standing, limited educational opportunities, and restricted access to information channels, migrants face significant disparities in healthcare utilization. Additionally, factors like unstable employment and transient living arrangements often hinder their access to adequate health education resources, creating further barriers to obtaining health services. Research indicates that migrant populations typically reside in unstable socioeconomic environments characterized by poor working conditions, substandard living quarters, and lower educational attainment. These circumstances heighten their susceptibility to chronic diseases and mental health issues (9, 10). More critically, migrants are more likely than residents to forgo essential healthcare services, which not only exacerbates their health risks (11, 12) but also widens health disparities (13–15).

Against this backdrop, to enhance access to basic public health services for migrant populations and improve their health outcomes, the Chinese government launched the Equalization of Basic Public Health and Family Planning Services for Migrant Populations initiative in 2013. This program aims to gradually narrow the health gap between migrants and residents by providing health education, establishing health records, and improving access to medical services. However, despite some achievements, significant inequalities persist in migrant populations’ access to public health services. This is particularly evident in areas such as health education, health records, family doctor contracts, and inpatient services, where service utilization rates among migrants remain consistently lower than those of residents. This indicates that equalization policies, while increasing service coverage for migrants, have not resolved underlying health inequalities, as evidenced by persistent barriers to access and disparities in health status. China's “Healthy China 2030 Plan Outline,” released in 2016, explicitly calls for promoting equalization of basic public health services and gradually narrowing health gaps between urban and rural areas, regions, and population groups. However, existing equalization policies still have limitations, particularly regarding health protection for the migrant population. Migrants not only face restrictions on public health services due to the household registration system but also grapple with inadequate social security and unstable employment. These factors make it difficult to effectively narrow the gap in access to and quality of health services between them and residents. Therefore, researching equity in migrant populations’ utilization of public health services has become particularly urgent. Equity analysis not only helps reveal shortcomings in existing policies’ impact on migrant health but also provides theoretical foundations for future policy adjustments. The inequality in health needs and service utilization among migrant populations is not only a matter of social justice but also a critical challenge in achieving universal health coverage.

Regarding the utilization of public health services and health status among the floating population, relevant research can be summarized in the following three aspects: First, the current status of public health service utilization and health among the floating population. Regarding the utilization of public health services by the migrant population, current research primarily analyzes the current status and influencing factors of their access to health education, health records, family doctor contracts, and outpatient and inpatient services. Findings consistently indicate that the utilization rate of basic public health services among the migrant population is relatively low (16). Factors such as marital status, educational attainment, household registration status, and migration distance significantly impact their utilization of basic public health services (17, 18). Regarding migrant health, existing research indicates that migrant populations face elevated health risks, necessitating increased attention to their health security issues (19, 20). Second, the equity in public health service utilization and health benefits. Existing research findings suggest that migrant populations’ utilization of public health services tends toward low-income groups, exhibiting a pro-poor bias (21, 22). Furthermore, the health concentration index for migrant populations is negative, indicating health inequities among migrants that correlate with income levels (23). Third, the health effects of public health service equalization policies. Current research has paid limited attention to the impact of different public health service utilization programs on migrant populations, focusing instead on using difference-in-differences models to examine the health effects of China's pilot basic public health service equalization policy. Findings indicate that this policy has a significant positive impact on migrant health. The pilot policy improves the overall health status of the migrant population and narrowes the health gap between migrants and the local population, but it also exacerbates health inequalities among the migrant population (24, 25).

The above studies demonstrate that scholars have paid extensive attention to the utilization of public health services and the health of the migrant population. However, there remains room for further expansion in related research. First, existing studies often examine the utilization and equity of only a single public health service for migrants, rather than providing a comprehensive analysis across multiple services. Second, current research pays insufficient attention to the relationship between migrant populations’ utilization of public services and their health outcomes. Finally, relevant studies also lack an analysis of the heterogeneity in how public health service utilization affects health.

Based on this analysis, this study utilizes nationally representative data from the China Migrant Dynamic Survey (CMDS) conducted by the National Health Commission. It focuses on the equity of public health service utilization among China's migrant population under pilot equalization policies. The research examines migrants’ access to services such as health education, health records, family doctor contracts, and inpatient care, assesses the fairness of their utilization of these services, and explores their impact on migrant health and health equity. The study will also conduct in-depth analyses from multiple dimensions (such as age, income, and education level) to examine how public health service utilization impacts health equity among different subgroups. This aims to provide valuable insights for improving the accessibility of public health services for the migrant population and enhancing their health levels. We further hope that these analyses will offer detailed recommendations for relevant policy formulation and optimization, thereby advancing the development of Healthy China.

Compared with existing research, the marginal contribution of this study lies in: First, a multidimensional analysis of equity in public health service utilization. Existing literature predominantly focuses on the utilization status and equity analysis of single public health service items (e.g., health education, health records) among migrant populations, lacking a comprehensive analysis of multiple public health services (health education, health records, family doctor contracts, and inpatient services) within this group. By conducting a holistic assessment of utilization across these four categories of public health services, this study provides more granular empirical evidence for the equalization of public health services. Second, analysis of heterogeneity in health equity. Existing research largely overlooks the heterogeneity of health equity across different groups, particularly the disparities between rural and urban migrant populations, as well as between low-income and high-income groups. By introducing multidimensional variables such as age, education level, and income, this study analyzes differences in public health service utilization among various migrant subgroups. Third, assessment of the health effects of public health equity policies. This study further evaluates the impact of China's public health service equalization policies on health equity among migrant populations. It reveals limitations in policy implementation, such as health education services failing to reduce health disparities among low-income groups while exacerbating the health advantages of high-income groups. Conversely, health records and family doctor contracting services have partially narrowed health gaps among migrants. These findings provide empirical evidence for refining public health service equalization policies. Fourth, the analysis of the relationship between health equity and health outcomes. Existing research has focused on analyzing the utilization rates of public health services and the health status of the migrant population. This study delves deeper into the relationship between public health service utilization and health outcomes, evaluating the specific impact of different service programs on the health of the migrant population. This provides new evidence for optimizing the structure of public health services. Fifth, the targeted and operational nature of the policy recommendations. Beyond its theoretical contributions, this study directly informs policy by proposing tailored health service strategies for different migrant subgroups. It proposes that future public health policies should prioritize the unique needs of migrant populations, refine service content and structure, thereby reducing health inequalities and advancing social equity.

## Theoretical framework

**Figure 1:**
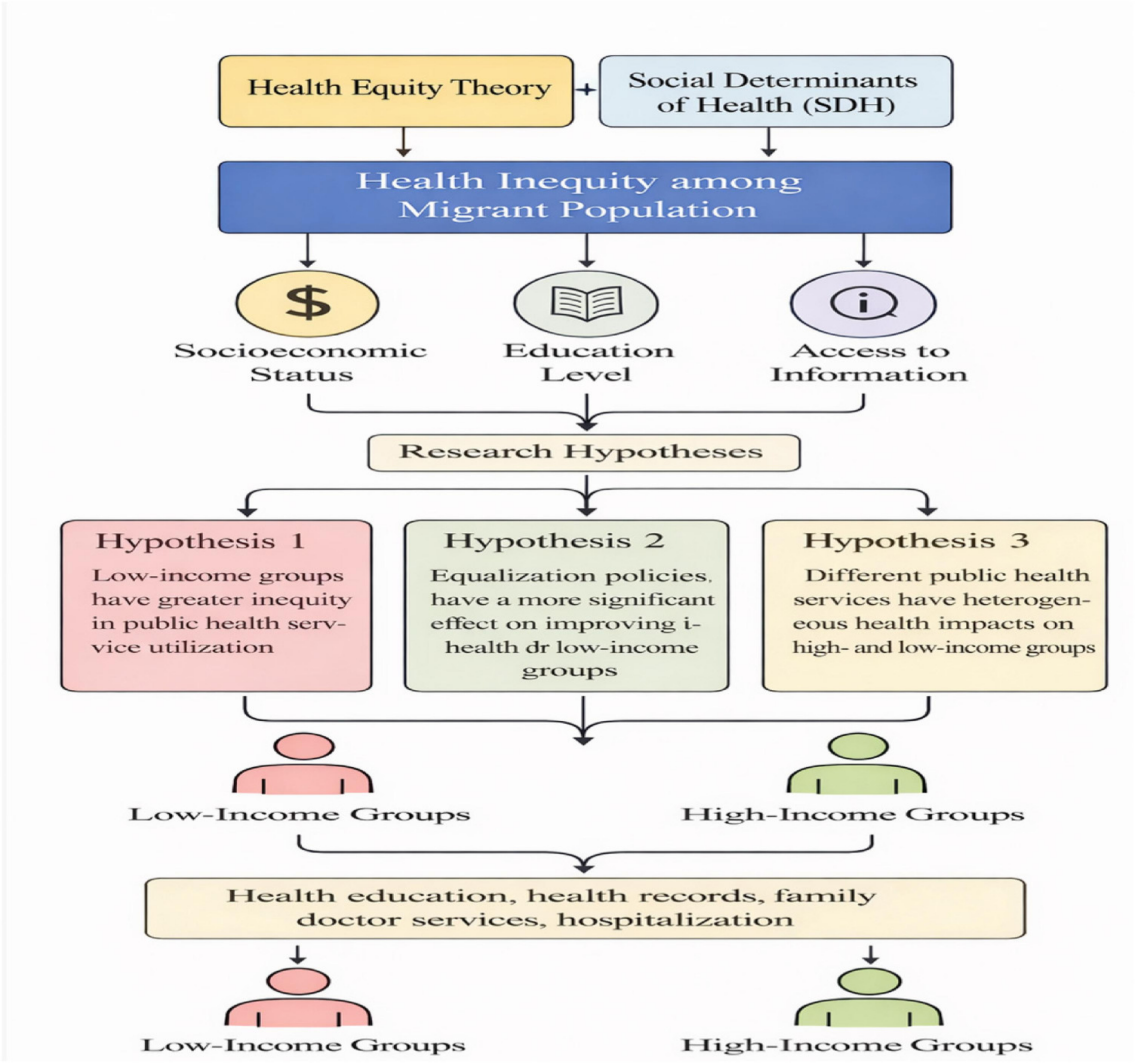
An analytical framework for migrant population public health service utilization and health equity.

This study draws upon health equity theory and the theory of social determinants of health to examine health inequalities among migrant populations and disparities in their utilization of public health services. It explores inequalities in public health service utilization among China's migrant populations and further analyzes the impact of these inequalities on health outcomes (Figure 1). Health equity theory posits that health inequalities primarily stem from inequities in social structures and institutions, particularly unequal access to health resources and services among different social groups (e.g., low-income populations, rural residents) (26, 27). This theory emphasizes that health is a social and institutional issue, and public health policies should reduce health disparities between social groups by improving the accessibility, universality, and equity of services (28, 29). The core tenet of this theoretical framework is that health status is influenced not only by individual biomedical factors but also by social determinants such as socioeconomic status, education, and environment. Therefore, achieving health equity requires not only narrowing disparities in access to health resources but also reducing gaps in health status across social groups. The theory of social determinants of health underscores that individual health is profoundly influenced not only by genetics and medical interventions but also by socioeconomic status, education, housing, employment, and social support (30, 31). According to the World Health Organization (WHO) definition, SDH encompasses socioeconomic status, educational attainment, employment status, social support, environmental conditions, health education, and other factors (32). These determinants not only influence individual health outcomes but also shape access to health services.

Migrant populations, as a distinct social group, face multiple health risks. Health inequalities among these groups primarily manifest as disparities in accessing public health services, including health education, health record establishment, family doctor contracts, and inpatient care. According to the aforementioned theories, health inequalities among migrant populations stem mainly from several factors. First, socioeconomic disparities: individuals with lower incomes often lack the financial means to secure high-quality medical services and may even forgo essential care due to cost. Second, geographic disparities: migrants often occupy unequal positions between urban and rural areas or across regions, preventing them from enjoying public health services and health security equivalent to local residents. Finally, institutional barriers: China's household registration system and social security framework impose restrictions on migrants, creating significant obstacles to accessing public health services—particularly foundational offerings like health education and family doctor contracts.

In this study, the integration of these two theories provides a comprehensive perspective: within the framework of health equity, social determinants such as socioeconomic status, education, and health literacy profoundly influence both health inequalities among migrant populations and disparities in their utilization of public health services. Guided by this theoretical framework, this study not only verifies the inequality in public health service utilization among the migrant population and analyzes the role of equalization policies in improving their health status, but also further examines the impact of different types of public health services (health education, health records, family doctor contracts, inpatient services, etc.) on health equity for the migrant population. This includes analyzing the differential effects across income groups and urban-rural populations. Based on health equity theory, this study proposes the following testable research hypotheses:

Hypothesis 1: Low-income groups experience greater inequality than high-income groups in accessing public health services (e.g., health education, health record establishment, family doctor contracts). This hypothesis is grounded in the core tenet of health equity theory that health inequalities are closely linked to socioeconomic status. Low-income groups typically face more systemic and economic barriers, leading to greater inequality in accessing public health services compared to high-income groups.
Hypothesis 2: Equalization policies for public health services yield more significant improvements in health outcomes for low-income groups. This hypothesis is rooted in the “service equalization” principle of health equity theory, positing that such policies will disproportionately benefit the health of low-income migrant groups, particularly in health education and records. Given the more urgent health needs of low-income groups, implementing equalization policies can help narrow the health gap between them and high-income groups.
Hypothesis 3: Health education services have a greater impact on the health of high-income groups, while establishing health records and family doctor contracts yield more significant health benefits for low-income groups. This hypothesis emphasizes the heterogeneity in the health impacts of different types of public health services across income groups. As a preventive service, health education may exert a greater impact on higher-income groups with better health status. Conversely, medical services such as health records and family doctor contracts may yield more pronounced health improvements for lower-income groups, who typically utilize these services less frequently.

## Research methodology

### Data source

We use data from the 2018 China Migrant Population Dynamics Surveillance Survey (CMDS), a program that is a nationally representative cross-sectional survey of the domestic migrant population conducted annually by China's National Health and Health Commission since 2009 (33). The CMDS is considered to be a good representative sample, as well as having a small sampling error (34), which adopts a hierarchical, multi-stage, and proportional-to-size PPS method for sampling (35). In the first stage, the townships (towns and streets) are sampled according to the PPS method. In the second stage, village (neighborhood) committees are selected within the selected townships (towns and streets) according to the PPS method. In the third stage, individual respondents are drawn within the selected village (neighborhood) committees. This survey also adopts strict methods to ensure data quality, including scientific design of questionnaires, training of enumerators, setting up survey supervisors to verify the questionnaires, and quality checking using telephone callbacks.

**Figure 2:**
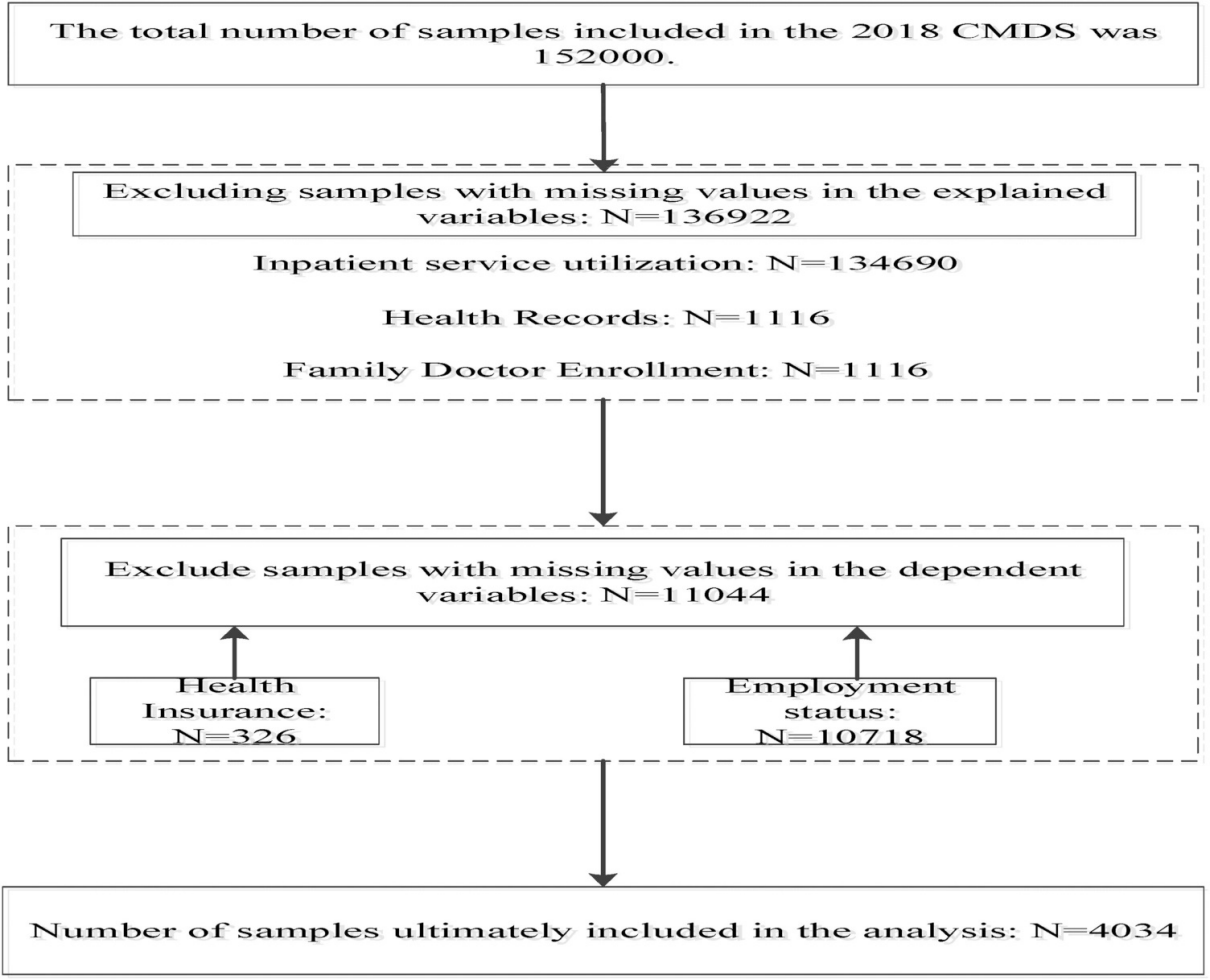
Flowchart of sample selection.

The CMDS targets migrant populations aged 15 and above, who have resided in their host locations for over one month and do not possess local household registration (hukou) at the district, county, or city level. The survey covers 31 provinces and the Xinjiang Production and Construction Corps in China. A total of 152,000 migrant samples are collected, providing a rich set of variables. The content of the CMDS survey covers basic demographic information, socioeconomic status, utilization of public health services and health care services, as well as the basic demographic information, social and economic conditions, and health care services. For the purposes of this study, the analytic sample was further restricted to respondents with valid information on the key variables used in the analysis, including public health service utilization, self-rated health, income, and other variables. Respondents with missing information on these variables were excluded. In addition, observations with implausible or outlying values identified during the data-cleaning process were removed. After applying these inclusion and exclusion criteria, the final analytic sample consisted of 4034 respondents. The sample selection process is presented in Figure 2.

It should be noted that this study is based on CMDS data and does not use permanent residents (non-migrant population) as a control group. This is primarily because these data themselves provide extensive information on migrant health service utilization. Additionally, the migrant population exhibits significant heterogeneity, and the primary focus of this study is to reveal health inequalities within the migrant group itself. Therefore, comparing migrant groups against permanent residents as a control group is not considered.

### Variable selection

#### Explained variables

The dependent variables in this study are public health service utilization and health status among migrants. Public health service utilization encompasses four aspects: health education, health records, family doctor contracts, and inpatient service utilization. These aspects are measured as follows: Health education is measured by the question “In the past year, have you received health education in the following aspects in your current place of residence or workplace?” The health education is measured using the question “Have you received health education in your current place of residence or organization in the past year in the following areas?” Measured using the question “Have you received health education in your current place of residence or organization in the past year in the following areas?” Measured using the question “Have you received health education in your current place of residence or organization in the past year in the following areas?” The health record is measured using the question “Have you established a local health record?”, with yes=1 and no = 0. The measurement question for the family doctor contract is “Have you contracted with a local family doctor?” The corresponding question for inpatient service utilization is “Have you personally been hospitalized in the last year?”, with responses coded as 1 for yes and 0 for no.

Self-assessed health has been used to measure the health status of the migrant population. Self-assessed health status has been reported to be consistent with the actual health level of the individual (36, 37), it serves as an indicator that can be applied across various contexts and can be used as a proxy for actual health status (38). The question is “What is your health status?”. The answer has four possible outcomes, which are not able to take care of yourself, unhealthy, but able to take care of yourself, basically healthy, and healthy. In this study, the four original outcomes are consolidated into a three-level ordinal measure of self-rated health: 1 = “unhealthy” (combining those who cannot care for themselves and those who are unhealthy but self-sufficient), 2 = “basically healthy”, and 3 = “healthy”. Thus, a higher score indicates better health.

#### Dependent variables

Based on the Andersen Healthcare Utilization Model (39, 40), previous research, and data availability, this study selects dependent variables to adjust for confounding effects. These variables are divided into three categories: propensity characteristics, enabling resources, and situational characteristics. Among them, propensity characteristics include gender, age, marital status, and education level (41). Enabling resources include household income level, household type, work status, and health insurance (42); and situational characteristics are mobility scope and reasons for mobility (43). According to the selected data conditions, the specific settings are as follows: gender is a dummy variable, 1 for males and 0 for females; age is the difference between the year of interview and the year of birth; marital status is a dummy variable, set to 1 if the individual is first married, remarried, or cohabitating, and 0 if the individual is unmarried, divorced, or widowed; and education is a variable with a scale of 0-4 based on the individual's academic qualifications, which are never been to school = 0, elementary school = 1, junior high school = 2, senior high school = 3, and university and above = 4; household income level is a data analysis that transforms data about household income into rankings within each province (<20th percentile, 20th-39th percentile, 40th-59th percentile, 60th-79th percentile, and ≥80th percentile); type of hukou is a dummy variable, with urban = 1 and rural = 0; and work status is also a dummy variable, with where unemployed=0, in-work=1; health insurance is categorized into Basic Medical Insurance System for Urban（BMISURR）and Basic Medical Insurance for Urban Employees（BMIUE）, which is in the BMISURR=1, BMIUE=2; mobility scope is assigned as follows: intercity=1, interprovince=2, intercounty=3; and mobility scope is assigned as follows: Mobility for Work Reasons=1, Mobility for Other Reasons=2, Mobility for Family Reasons=3. Considering that provinces differ in their economic and social environments, which may influence migrants’ public health service utilization and health outcomes (44, 45). In this study, the provinces where the migrant population resides are also divided into different regions, including the East, the Central, and the West, which are coded as 3, 2, and 1. Combined with the above analysis, the definition and assignment of variables in this study are shown in Table 1.

**Table 1:** Definition of variables.

Variables		Definition
Public health services utilization	Health education	No = 0; Yes = 1
	Health record	No = 0; Yes = 1
	Family doctor contracting	No = 0; Yes = 1
	Utilization of inpatient services	No = 0; Yes = 1
Health		Unhealthy = 1; Basically healthy = 2; Healthy = 3
Gender		Female = 0; Male = 1
Age		15–30 = 1; 31–45 = 2; 46–60 = 3; 61+=4
Education level		Illiteracy = 1; Elementary school = 2; Middle school = 3; High school = 4; University = 5
Marriage		Unmarried = 0; Married = 1
Working situation		Not worked = 0; worked = 1
Household registration		Rural = 1; Urban = 2
Range of mobility		Cross-county = 1; cross-city = 2; cross-province = 3
Reasons for mobility		Family = 1; Work = 2; Other = 3
Household income		Lowest(<percentile20) = 1
		Lower(percentile20–39) = 2
		Middle(percentile40–59) = 3
		Higher(percentile60–79) = 4
		Highest(≥percentile80) = 5
Health insurance		BMISURR=1; BMIUE=2
Region		West = 1; Central = 2; East = 3

### Statistical models

#### Equity in public health service utilization

The Concentration Index (CI) is widely used to measure income-related inequalities in health-related fields (46, 47). The calculation formula is as follows:

$$\mathrm{CI}=2\mathrm{cov}(\;y_{i\;},R_{i})/\mu$$

Where $y_{i}$ represents the outcome variable of public health service utilization, μ represents the average level of the variable in the population, and $R_{i}$ represents the fractional rank of sample *i* in the income distribution. The value of CI ranges from (−1,1), CI > 0 indicates that there is a pro-rich inequality in the outcome variable, and CI < 0 suggests that there is a pro-poor inequality in the outcome variable. The larger the absolute value of the CI, the more sensitive the outcome variable is to income level, and the greater the degree of inequality (48).

Since all outcome variables in this study are binary variables, the modified concentration index (Erreygers concentration index, EI) is used to evaluate the equity in the utilization of public health services for the migrant population (49, 50), and the calculation is publicized as:

$$\mathrm{EI}=\frac{4\mu }{\left(y_{\max}-y_{\min}\right)}\mathrm{CI}(y)$$

where, $y_{\max}$ and $y_{\min}$ are the maximum and minimum values of the public health services and health variables, respectively, and CI(y) is the CI of y.

The CI decomposition allows for the decomposition of the CI for public health service utilization and health into the contribution of each influencing factor to the inequality of the outcome variable, where the contribution of each factor is obtained by multiplying the elasticity of the outcome variable by respect to that factor with the degree of inequality of that factor concerning income (51). The decomposition allows for further exploration of the impact of each influencing factor on the inequality of the dependent variables, and the CI decomposition is based on a general regression analysis with the following formula:

$$C=\sum k\left(\frac{\beta_{k\;\;{\bar{x}}_{k}\;\;\;}}{\mu }\right)C_{K}+\frac{GC_{\varepsilon }}{\mu }$$

Where C is the CI of the explanatory variable, μ is the mean of public health service utilization (y), $\beta_{k}$ denotes the coefficients of the logit regression, ${\bar{x}}_{k}$ denotes the mean of the Kth variable, $C_{K}$ is the CI of the Kth variable, and $GC_{\varepsilon }$ is the generalized CI of the residual term. However, when the outcome indicator is a dichotomous variable, the decomposition of the EI is multiplied by 4µ on top of the CI decomposition, i.e:

$$EI_{y}=4\left(\sum k\beta_{k}\;{\bar{x}}_{k}C_{k}+GC_{\varepsilon }\right)$$

### Health inequality and decomposition

The Health Concentration Index (HCI) is a commonly used indicator to measure the level of health inequality. This study uses self-rated health status as the basis for calculating the health concentration index. The original data of the self-assessed health level is an ordered categorical variable with values of three categories, and if the self-assessed health level is transformed into a binary variable with values of 0 and 1, different truncation points of 0 and 1 values will yield different results in the calculation of the health concentration index (52, 53). Therefore, this paper draws on the approach of van Doorslaer and Jones (54) and uses the Ordered Probit model to transform the self-assessed health level into continuous values in the interval [0, 1]. The specific method is as follows: the original data of self-assessed health level takes the values of 1, 2, and 3, and the corresponding Ordered Probit model is established as:

$$\begin{array}{ccc} h_{it\;}=j, & \gamma_{\;j-1}<h_{it}^{*}\leq \gamma_{j}, & \;j=1,\;2,\;3 \end{array}$$

In this formula, *hit* is an ordered categorical variable representing the self-assessed health level. Individual *i*, *γ j* is the cut point to be estimated, and $h_{it}^{*}$ is a potential continuous variable behind *hit*. This can be expressed as an equation for a series of variables such as an individual's demographic characteristics（$X_{it\;}$）as follows:

$$h_{it}^{*}=\beta_{1\;}X_{it\;}+\varepsilon_{it\;},\varepsilon_{it\;∼}N(0,1)$$

The probability that *hit* takes the value of *j* can be obtained from the assumption of the distribution of the disturbance term $\varepsilon_{it}$ in the above equation:

$$P_{ij}=P(h_{\mathrm{it}}=j)=\theta (\gamma_{i}-\beta_{1}X_{\mathrm{it}})-\theta (\gamma_{i-1}-\beta_{2}X_{\mathrm{it}})$$

θ(·) in the formula is the cumulative density function of the standard normal distribution. This study establishes the log-likelihood function of the Ordered Probit model and uses maximum likelihood estimation to obtain the estimated value of coefficient β and tangent point $\gamma_{i}$. Then, according to the above formula, we calculate the linear estimated value of the latent variable of self-assessment of health level $Sah_{it\;}$, which takes the range of values of (-∞,∞). This study converts the predicted value into a continuous self-assessed health level score E in the interval [0, 1] according to the treatment of standardization of deviation. Based on the continuous self-assessed health level score determined above, the health concentration index, which is expressed as two times the area between the health concentration curve and the fairness line, is derived. This paper draws on Wagstaff et al. (54), the expression for the health concentration index is defined as:

$$\mathrm{CI}=\frac{2}{H}\mathrm{COV}(Sah_{i},R_{i})=\frac{2}{\mathrm{nH}}\sum_{i=1}^{n}Sah_{i}R_{i}-1$$

The CI represents the health concentration index, which ranges from −1 to 1. The larger the value of the self-assessed health level chosen in this paper, the better the health of the individual. In this paper, the larger the value of the self-assessed health level $Sah_{it\;}$, the better the health status of the individual. So when the CI is positive, it indicates that individuals with higher incomes are in better health, and there is pro-rich health inequality. H is the average health status of the sample, and *Ri* is the quartile value of the ith person in the sample ranked in descending order of income, which is calculated by(*i* - 0.5)/*n*.

This paper calculates concentration indices and elasticities for each influencing factor to decompose health inequality and identify each factor's contribution to health inequality. The contribution of each factor to health inequality is related both to its direct impact on health (measured by elasticity) and to the indirect impact of income on health inequality through that factor (measured by concentration index). The decomposition of health inequality starts by estimating a regression model on migrant health determinants to obtain each factor's coefficient. The specific model is set up as follows:

$$Sah_{it\;}=\alpha_{0}=+\alpha_{1}X_{it}+\varepsilon_{it}$$

where $Sah_{it\;}$ is the health level of individual *i* at time *t*, $X_{it}$ is the demographic and other characteristics of individual *i* at time *t*, and $\varepsilon_{it}$ is the random error term.

In this paper, we calculate the corresponding elasticities using the means of each factor, and then derive the concentration index for each factor, and then use the elasticities as weights for weighted averages to achieve the decomposition of health inequality, i.e:

$$\mathrm{CI}=\underset{k}{\sum }n_{K}CI_{K}+\frac{GC_{\varepsilon }}{\mu }$$

CI represents the health concentration index. $CI_{K}$ is the concentration index for the kth influencing factor, representing the indirect effect of each factor on health inequality as a result of the influence of income. $n_{K}$ represents the elasticity of demand for health for the kth factor, and $\frac{GC_{\varepsilon }}{\mu }$ is the effect of the disturbance term on the health concentration index.

### Statistical analysis

The stata22.0 software is used to analyze the data. First, descriptive statistics are used to analyze the data distribution of the explained and dependent variables; then the 2-test is used to analyze the differences in public health service utilization and health level of the migrant population under different characteristics. Second, variables that are statistically significant in the univariate analysis are included in the logistic regression model to analyze the influencing factors of public health service utilization and health. Third, concentration indices and curves are calculated for the migrant population's public health service utilization and health status; the contributions of various factors to equity in these outcomes are then decomposed. Fourth, the heterogeneity of the impact of public health education on health equity of the migrant population is analyzed in four dimensions: urban and rural, age, education, and income.

## Research results

### Characteristics of respondents

Table 2 demonstrates the results of descriptive statistics of the main variables in this study. Among the 4,034 respondents, 64.3% rate their health as “healthy” and 24.8% as “basically healthy.” The percentage of the migrant population receiving health education is 82.10%, but 17.90% of the migrant population do not receive health education. Only 35.5% of migrants have established health records, and merely 16.3% have signed contracts with local family doctors. The proportion receiving hospitalization service utilization is 31.1%; the education level of the migrant population is generally low, with the proportion of those who have a university degree or higher being only 38.47%, while the proportion of those who have a high school degree or lower being 61.53%. 53.54% of the migrant population is of rural household registration, and only 46.46% of the migrant population is from urban areas. Analyzing the scope of mobility, the proportion of inter-provincial mobility is the highest, followed by intra-provincial inter-city and intra-city inter-county mobility. The majority of the migrant population has an income below the medium level (57.58%). Regarding health insurance coverage, 65.37% of the migrant population are enrolled in the Basic Medical Insurance for Urban Employees (BMIUE), and 34.63% in the Basic Medical Insurance for Urban and Rural Residents (BMISURR). In terms of regional distribution, the proportions of the migrant population from the east, central, and west are 46.1%, 22.8%, and 31.1% respectively.

**Table 2:** Basic characteristics of the samples.

Variables		n	%
Health	Unhealthy	439	10.9
	Basically healthy	1002	24.8
	Healthy	2593	64.3
Health education	No	722	17.9
	Yes	3312	82.1
Health record	No	2601	64.5
	Yes	1433	35.5
Family doctor contracting	No	3376	83.7
	Yes	658	16.3
Utilization of inpatient services	No	2778	68.9
	Yes	1256	31.1
Gender	Female	2279	56.49
	Male	1755	43.51
Age	15–30	1197	29.67
	31–45	1839	45.59
	46–60	759	18.82
	61+	239	5.92
Education level	Illiteracy	134	3.32
	Elementary school	517	12.82
	Middle school	1051	26.05
	High school	780	19.34
	University	1552	38.47
Marriage	Unmarried	677	16.78
	Married	3357	83.22
Working situation	Not worked	825	20.45
	Worked	3209	79.55
Household registration	Rural	2160	53.54
	Urban	1874	46.46
Range of mobility	Cross-county	501	12.42
	Cross-city	1592	39.46
	Cross-province	1941	48.12
Reasons for mobility	Family	665	16.48
	Work	3303	81.88
	Other	66	1.64
Household income	Lowest(<percentile20)	855	21.19
	Lower(percentile20–39)	730	18.10
	Middle(percentile40–59)	738	18.29
	Higher(percentile60–79)	805	19.96
	Highest(≥percentile80)	906	22.46
Health insurance	BMISURR	2637	65.37
	BMIUE	1397	34.63
Region	West	1254	31.1
	Central	920	22.8
	East	1860	46.1

### Analysis of differences in basic public health service utilization and health status among migrants with different characteristics

Table 3 presents the analysis of the differences in the utilization of public health services and the health level of the migrant population under different characteristics. For health education, statistically significant differences (all *P* < 0.05) are found among migrants with different ages, education levels, marital status, employment status, household registration, migration scope, migration reasons, income levels, insurance types, and regional distributions. In terms of the health record, the differences in the health of the migrant population are statistically significant in terms of gender, age. In terms of health records, the differences in the health of migrant populations by gender, age, education level, marriage, job, mobility range, reason for mobility, income, type of insurance participation, and region are statistically significant (all *P* < 0.05). For family doctor contracts, the differences in the health of migrant populations by gender, age, education level, marriage, job, mobility range, reason for mobility, income, type of insurance participation, and region are statistically significant (all *P* < 0.05). In terms of inpatient service utilization, the differences in the health of migrant populations by gender, age, education, marriage, job, mobility range, reason for mobility, income, type of insurance participation, and region are statistically significant (all *P* < 0.05). Regarding health status, significant differences (all *P* < 0.05) are observed across different genders, ages, education levels, marital status, employment status, household registration, migration scope, migration reasons, income levels, insurance types, and regions.

**Table 3:** Differential analysis of the utilization of public health services and health level of mobile population under different characteristics.

Variables		Health education				Health record				Family doctor contracting				Utilization of inpatient services				Health					
		Yes		No		Yes		No		Yes		No		Yes		No		Unhealthy		Basically healthy		Healthy	
		n	%	n	%	n	%	n	%	n	%	n	%	n	%	n	%	n	%	n	%	n	%
Gender	Female	401	55.54	1878	56.70	1406	54.06	837	60.92	1877	55.60	402	61.09	1045	58.58	874	69.69	249	56.72	526	52.50	1504	58.00
	Male	321	44.46	1434	43.30	1195	45.94	560	39.08	1499	44.40	256	38.91	1373	49.42	382	30.41	190	43.28	476	47.50	1089	42.00
	χ^2^	0.3261				17.717				6.767				127.175				8.9288					
	P value	0.568				0.000				0.009				0.000				0.012					
Age	15–30	169	23.41	1028	29.67	815	31.33	382	26.66	1020	30.21	177	26.90	793	28.55	404	32.17	13	2.96	179	17.86	1005	38.76
	31–45	302	41.83	1537	45.59	1225	47.10	614	42.85	1573	46.59	266	40.43	1305	46.98	534	42.52	86	19.59	481	48.00	1272	49.06
	46–60	189	26.18	570	18.82	452	13.38	307	21.42	623	18.45	136	20.67	539	19.40	220	17.52	205	46.70	263	26.25	291	11.22
	61+	62	8.59	177	5.92	109	4.19	130	9.07	160	4.74	79	12.01	141	5.08	98	7.80	135	30.75	79	7.88	25	0.96
	χ^2^	50.217				55.666				57.149				20.087				1159.153					
	P value	0.000				0.000				0.000				0.000				0.000					
Education level	Illiteracy	40	5.54	94	2.84	74	2.85	60	4.19	104	3.08	30	4.56	95	3.42	39	3.11	79	18.00	35	3.49	20	0.77
	Elementary school	129	17.87	388	11.71	308	11.84	209	14.58	398	11.79	119	18.09	336	12.10	181	14.41	173	39.41	166	16.57	178	6.86
	Middle school	194	26.87	867	25.88	650	24.99	401	27.98	865	25.62	186	28.27	726	26.13	325	25.88	125	28.47	315	31.44	611	23.56
	High school	104	14.40	676	20.41	487	18.72	293	20.45	667	19.76	113	17.17	548	19.73	232	18.47	46	10.48	187	18.66	547	21.10
	University	225	35.32	1297	39.16	1082	41.60	470	32.80	1342	39.75	210	31.91	1073	38.62	479	38.14	16	3.64	299	29.84	1237	47.71
	χ^2^	44.093				33.632				32.827				4.657				916.0474					
	P value	0.000				0.000				0.000				0.324				0.000					
Marriage	Unmarried	114	15.79	563	17.00	477	18.34	200	19.36	591	17.51	86	13.07	581	20.91	96	7.64	53	12.07	145	14.47	479	18.47
	Married	608	84.21	2749	83.00	2124	81.66	1233	86.04	2785	82.49	572	86.93	2197	79.09	1160	92.36	386	87.93	857	85.53	2114	81.53
	χ^2^	0.621				12.706				7.759				109.075				16.1102					
	P value	0.431				0.000				0.000				0.000				0.000					
Working situation	Not worked	191	26.45	634	19.14	451	17.34	374	26.10	614	18.19	211	32.07	416	14.97	409	32.56	271	61.73	217	21.66	337	13.00
	Worked	531	73.55	2678	80.86	2150	82.66	1059	73.90	2762	81.81	447	67.93	2362	85.03	847	67.44	168	38.27	785	78.34	2256	87.00
	χ^2^	19.479				43.578				65.208				164.479				549.2939					
	P value	0.000				0.000				0.000				0.000				0.000					
Household registration	Rural	410	56.79	1750	52.84	1381	53.09	779	54.36	1795	53.17	365	55.47	1505	54.18	655	52.15	325	74.03	561	55.99	1274	49.13
	Urban	312	43.21	1562	47.16	1220	46.91	654	45.64	1581	46.83	293	44.53	1273	45.82	601	47.85	114	25.97	441	44.01	1319	50.87
	χ^2^	3.716				0.596				1.173				1.427				96.7766					
	P value	0.054				0.440				0.279				0.232				0.000					
Range of mobility	Cross-county	73	10.11	428	12.92	303	11.65	198	13.82	400	11.85	101	15.35	308	11.09	193	15.37	57	12.98	150	14.97	294	11.34
	Cross-city	257	35.60	1335	40.31	955	36.72	637	44.45	1284	38.03	308	46.81	1027	37.97	565	44.98	202	46.01	354	35.33	1036	39.95
	Cross-province	392	54.29	1549	46.77	1343	51.63	598	41.73	1692	50.12	249	37.84	1443	51.94	498	39.65	180	41.00	498	49.70	1263	48.71
	χ^2^	14.084				36.339				39.429				54.002				22.4012					
	P value	0.001				0.000				0.000				0.000				0.000					
Reasons for mobility	Family	139	19.25	526	15.88	383	14.73	282	19.68	498	14.75	167	25.38	387	13.93	278	22.13	137	31.21	172	17.17	356	13.73
	Work	572	79.22	2731	82.46	2184	83.97	1119	78.09	2834	83.95	469	71.28	2354	84.74	949	75.56	278	63.33	807	80.54	2218	85.54
	Other	11	1.52	55	1.66	34	1.31	32	2.23	44	1.30	22	3.34	37	1.33	29	2.31	24	5.47	23	2.30	19	0.73
	χ^2^	4.909				22.497				62.541				49.253				147.8360					
	P value	0.086				0.000				0.000				0.000				0.000					
Household income	Lowest (<percentile20)	195	27.01	660	19.93	510	19.61	345	24.08	671	19.88	184	27.96	567	20.41	288	22.93	240	54.67	249	24.85	366	14.11
	Lower (percentile20–39)	125	17.31	605	18.27	466	17.92	264	18.42	588	17.42	142	21.58	490	17.64	240	19.11	90	20.50	202	20.16	438	16.89
	Middle (percentile40–59)	125	17.31	613	18.51	457	17.57	281	19.61	629	18.63	109	16.57	491	17.67	247	19.67	56	12.76	180	17.96	502	19.36
	Higher (percentile60–79)	125	17.31	680	20.53	518	19.92	287	20.03	679	20.11	126	19.15	555	19.98	250	19.90	37	8.43	183	18.26	585	22.56
	Highest (≥percentile80)	152	21.05	754	22.77	650	24.99	256	17.86	809	23.96	97	14.74	675	24.30	231	18.39	16	3.64	188	18.76	702	27.07
	χ^2^	18.635				31.827				44.662				18.934				453.9616					
	P value	0.001				0.000				0.000				0.001				0.000					
Health insurance	BMISURR	406	56.23	2231	67.35	813	31.26	584	40.75	1078	31.93	319	48.48	883	31.79	514	40.92	84	80.87	598	59.68	1955	75.40
	BMIUE	316	43.77	1081	32.65	1788	68.74	849	59.25	2298	68.07	339	51.52	1895	68.21	742	59.08	355	19.13	404	40.32	638	24.60
	χ^2^	32.428				36.808				66.619				31.905				544.0058					
	P value	0.000				0.000				0.000				0.000				0.000					
Region	West	166	22.99	1088	32.85	757	29.10	497	34.68	1007	29.83	247	37.54	801	28.83	453	36.07	188	48.82	366	36.53	700	27.00
	Central	174	24.10	746	22.52	510	19.61	410	28.61	715	21.18	205	31.16	585	21.06	335	26.67	173	39.41	244	24.53	503	19.40
	East	382	52.91	1478	44.63	1334	51.29	526	36.71	1654	48.99	206	31.31	1392	50.11	468	37.26	78	17.77	392	39.12	1390	53.61
	χ^2^	27.999				84.698				71.917				57.469				228.9564					
	P value	0.000				0.000				0.000				0.000				0.000					

### Analysis of equity in public health service utilization among migrants

#### Concentration index of public health service utilization

**Figure 3:**
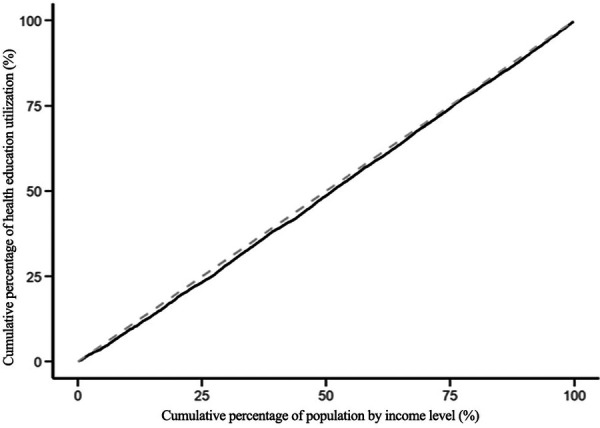
Concentration curve of health education service utilization.

**Figure 4:**
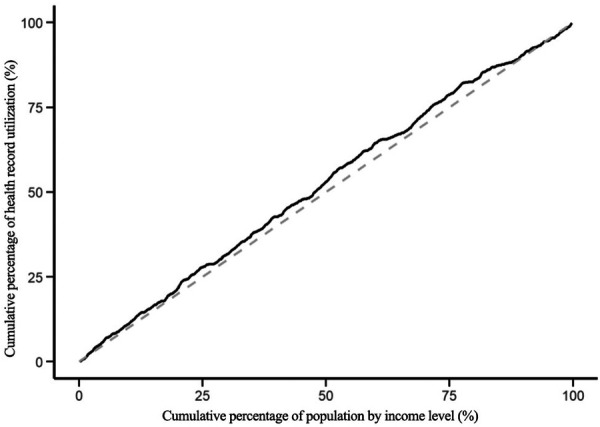
Centralized curve for the establishment of health records.

**Figure 5:**
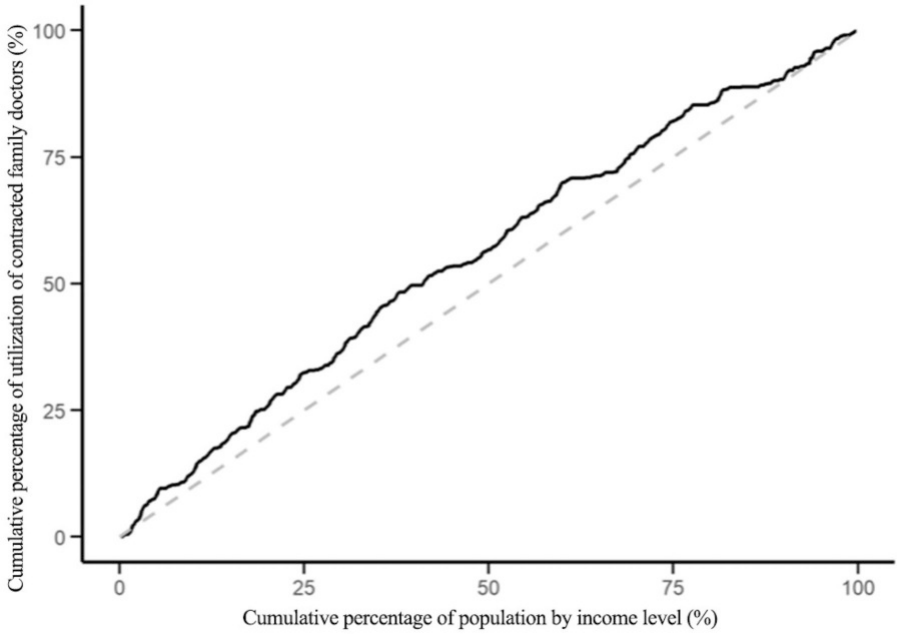
Concentration curve of contracted family doctor services.

**Figure 6:**
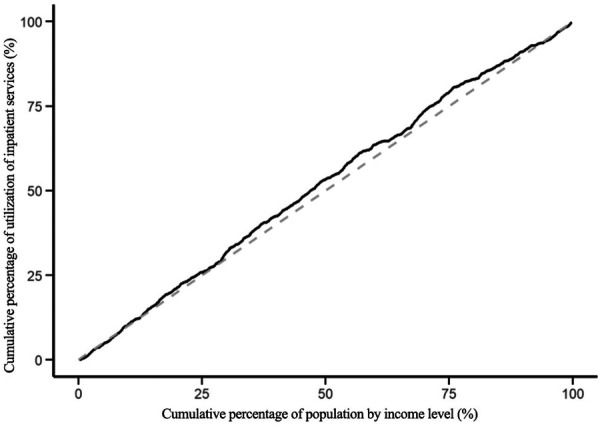
Concentration curve for utilization of inpatient services.

The modified concentration indices for the utilization of health education, health records, family doctor contracting, and hospitalization services for the migrant population are 0.045, −0.085, −0.084 and −0.062 respectively. The positive Erreygers Concentration Index for health education utilization indicates a pro-rich inequality. This is visualized in Figure 3, where the concentration curve lies below the line of equality. The modified concentration indices for the utilization of health records, family doctor contracting and hospitalization services are all less than 0, and the concentration curves are above the absolute fairness line, indicating that these three services are inclined to low-income migrant populations, see Figures 4–6.

#### Decomposition results of concentration index for public health service utilization among migrants

Taking the statistically significant variables in the single-factor analysis in Table 3 as independent variables, the health education, health records, family doctor contracting, and hospitalization service utilization of the migrant population as dependent variables respectively, we analyze the influencing factors of the migrant population's public health service utilization and further explore the degree of contribution of different factors. The analysis results are shown in Table 4.

**Table 4:** Decomposition of factors influencing the utilization of public health services by the mobile population and the correction index.

Variables		Health education								Health record							
		β	OR	95%CI	P	Coefficient of elasticity	EI	Degree of contribution	Contribution rate（%）	β	OR	95%CI	P	Coefficient of elasticity	EI	Degree of contribution	Contribution rate（%）
Gender	Female (reference)																
	Male	—	—	—	—	—	—	—	—	−0.289	0.749	0.647—0.867	0.000	−0.345	0.035	−0.007	8.100
Age	15–30 (reference)																
	31–45	−0.165	0.847	0.684—1.051	0.132	0.103	0.01	0.001	1.200	0.074	1.077	0.909—1.276	0.390	−0.221	0.01	−0.001	1.400
	46–60	−0.617	0.539	0.408—0.714	0.000	−0.122	−0.199	0.032	72.300	0.302	1.352	1.069—1.708	0.012	0.137	−0.199	−0.036	42.800
	61+	−0.435	0.647	0.429—0.975	0.037	−0.037	−0.11	0.017	38.000	0.729	2.074	1.449—2.968	0.000	0.138	−0.11	−0.064	75.000
Education level	Illiteracy (reference)																
	Elementary school	0.184	1.202	0.775—1.864	0.410	−0.077	−0.222	0.033	74.500	0.143	1.154	0.766—1.737	0.494	0.087	−0.222	−0.037	44.100
	Middle school	0.429	1.536	0.984—2.396	0.059	−0.016	−0.214	0.003	7.400	0.326	1.385	0.919—2.086	0.119	0.113	−0.214	−0.023	27.200
	High school	0.679	1.971	1.216—3.197	0.006	0.099	0.006	0.001	1.800	0.441	1.554	1.009—2.393	0.045	0.06	0.006	<0.001	−0.600
	University	0.260	1.268	0.792—2.125	0.301	0.077	0.503	0.025	56.200	0.214	1.239	0.799—1.921	0.337	−0.409	0.503	−0.134	1.577
Marriage	Unmarried (reference)																
	Married	—	—	—	—	—	—	—	—	0.134	1.143	0.932—1.401	0.198	0.762	−0.23	−0.053	62.000
Working situation	Not worked (reference)																
	Worked	0.169	1.183	0.934—1.501	0.163	0.405	0.241	0.031	68.500	−0.157	0.855	0.701—1.044	0.124	−1.167	0.241	−0.088	1.040
Household registration	Rural (reference)																
	Urban	0.011	1.010			0.09	0.246	0.012	26.700	—	—	—	—	—	—	—	—
Range of mobility	Cross-county (reference)																
	Cross-city	−0.147	0.863	0.645—1.154	0.321	0.096	−0.023	−0.001	−3.100	0.099	1.105	0.888—1.373	0.370	0.357	−0.023	−0.005	6.100
	Cross-province	−0.270	0.763	0.569—1.022	0.071	−0.177	0.064	−0.006	−13.000	−0.128	0.880	0.702—1.103	0.267	−0.541	0.064	−0.018	21.00
Reasons for mobility	Family (reference)																
	Work	0.102	1.107	0.871—1.406	0.405	0.209	0.157	0.01	22.400	−0.033	0.968	0.793—1.181	0.746	−0.887	0.157	−0.043	50.200
	Other	0.428	1.534	0.762—3.091	0.231	0.002	−0.018	<0.001	−1.000	0.154	1.167	0.671—2.031	0.584	0.025	−0.018	−0.007	7.900
Household income	Lowest(<percentile20) (reference)																
	Lower (percentile20–39)	0.198	1.217	0.936—1.581	0.142	0.014	−0.286	−0.006	−12.700	−0.104	0.901	0.724—1.121	0.348	0.017	−0.286	−0.007	8.100
	Middle (percentile40–59)	0.151	1.163	0.887—1.525	0.276	0.018	−0.023	−0.001	−1.300	0.106	1.112	0.887—1.394	0.354	0.069	−0.023	−0.002	2.600
	Higher (percentile60–79)	0.192	1.216	0.917—1.614	0.173	0.051	0.280	0.018	40.000	0.031	1.032	0.818—1.301	0.798	0.004	0.28	0.001	−1.600
	Highest (≥percentile80)	0.055	1.062	0.796—1.418	0.682	0.027	0.697	0.021	47.400	−0.195	0.823	0.645—1.049	0.116	−0.27	0.697	−0.209	2.461
Health insurance	BMISURR (reference)																
	BMIUE	0.453	1.579	1.274—1.957	0.000	0.377	0.377	0.054	1.213	−0.047	0.954	0.799—0.138	0.600	−0.762	0.377	−0.11	1.293
Region	West (reference)																
	Central	−0.542	0.579	0.455—0.739	0.000	−0.024	−0.07	0.002	4.200	0.195	1.215	1.011—1.459	0.038	0.319	−0.07	−0.025	28.900
	East	−0.873	0.418	0.332—0.525	0.000	−0.187	0.191	−0.019	−43.100	−0.349	0.705	0.593—0.839	0.000	−0.774	0.191	−0.08	94.40
Variables		Family doctor contracting								Utilization of inpatient services							
		β	OR	95%CI	P	Coefficient of elasticity	EI	Degree of contribution	Contribution rate（%）	β	OR	95%CI	P	Coefficient of elasticity	EI	Degree of contribution	Contribution rate（%）
Gender	Female (reference)																
	Male	−0.085	0.918	0.760—1.109	0.377	−0.604	0.035	−0.012	14.300	−0.619	0.538	0.461—0.629	0.000	−1.124	0.035	−0.022	0.361
Age	15–30 (reference)																
	31–45	−0.067	0.935	0.743—1.177	0.567	−0.702	0.01	−0.004	4.300	−0.449	0.638	0.532—0.766	0.000	−0.264	0.01	−0.001	0.022
	46–60	−0.063	0.938	0.694—1.268	0.679	0.163	−0.199	−0.043	51.000	−0.528	0.590	0.459—0.758	0.000	−0.076	−0.199	0.02	−0.323
	61+	0.400	1.492	1.001—2.227	0.050	0.366	−0.11	−0.17	2.012	−0.256	0.774	0.535—1.119	0.174	0.087	−0.11	−0.04	65.200
Education level	Illiteracy (reference)																
	Elementary school	0.298	1.348	0.836—2.175	0.221	0.394	−0.222	−0.171	2.024	0.605	1.833	1.175—2.859	0.000	0.083	−0.222	−0.036	0.580
	Middle school	0.306	1.358	0.835—2.209	0.217	0.215	−0.214	−0.044	52.200	0.658	1.931	1.234—3.022	0.004	−0.011	−0.214	0.002	−0.037
	High school	0.195	1.216	0.723—2.042	0.461	−0.204	0.006	−0.002	0.020	0.693	2.001	1.251—3.201	0.004	−0.05	0.006	0	0.007
	University	0.329	1.389	0.820—2.354	0.221	−0.806	0.503	−0.264	3.127	0.831	2.296	1.426—3.695	0.001	−0.025	0.503	−0.008	13.400
Marriage	Unmarried (reference)																
	Married	0.183	1.200	0.914—1.576	0.189	1.758	−0.23	−0.121	1.440	1.336	3.802	2.956—4.890	0.000	3.105	−0.23	−0.214	3.456
Working situation	Not worked (reference)																
	Worked	−0.175	0.839	0.661—1.065	0.151	−3.672	0.241	−0.278	3.297	−0.837	0.433	0.353—0.529	0.000	−2.577	0.241	−0.195	3.144
Household registration	Rural (reference)																
	Urban	—	—	—	—	—	—	—	—	—	—	—	—	—	—	—	—
Range of mobility	Cross-county (reference)																
	Cross-city	0.008	1.008	1.008	0.950	0.872	−0.023	−0.013	14.900	−0.182	0.834	0.667—1.041	0.108	0.421	−0.023	−0.006	9.800
	Cross-province	−0.305	1.737	0.737	0.031	−1.478	0.064	−0.049	57.800	−0.445	0.641	0.508—0.807	0.000	−0.769	0.064	−0.025	40.900
Reasons for mobility	Family (reference)																
	Work	−0.336	0.714	0.714	0.004	−3.741	0.157	−0.18	2.133	0.029	1.029	0.841—1.261	0.777	−1.54	0.157	−0.074	1.193
	Other	0.191	1.210	1.210	0.510	0.097	−0.018	−0.026	30.800	0.225	1.253	1.715—2.194	0.431	0.029	−0.018	−0.008	0.128
Household income	Lowest(<percentile20) (reference)																
	Lower (percentile20–39)	0.093	1.098	0.847—1.423	0.482	0.295	−0.286	−0.117	1.383	0.033	1.034	0.824—1.299	0.770	0.057	−0.286	−0.023	36.300
	Middle (percentile40–59)	−0.162	0.851	0.641—1.128	0.206	−0.16	−0.023	0.005	−5.900	0.176	1.192	0.943—1.507	0.141	0.077	−0.023	−0.002	39.000
	Higher (percentile60–79)	−0.042	0.959	0.719—1.278	0.774	−0.075	0.28	−0.026	31.100	0.047	1.048	0.822—1.337	0.705	−0.003	0.28	−0.001	17.000
	Highest (≥percentile80)	−0.356	0.701	0.509—0.963	0.028	−0.827	0.697	−0.641	7.601	0.004	1.004	0.776—0.298	0.973	−0.255	0.697	−0.198	3.188
Health insurance	BMISURR (reference)																
	BMIUE	−0.297	0.742	0.595—0.926	0.008	−2.79	0.377	−0.402	4.768	−0.098	0.906	0.752—1.092	0.301	−0.833	0.377	−0.12	1.934
Region	West (reference)																
	Central	1.141	1.152	0.926—1.433	0.204	0.729	−0.07	−0.056	66.500	−0.123	0.883	0.729—1.069	0.204	0.227	−0.07	−0.017	28.200
	East	−0.336	0.714	0.571—0.895	0.003	−2.107	0.191	−0.218	2.588	−0.350	0.704	0.587—0.845	0.000	−0.778	0.191	−0.081	1.299

“-” indicates that this variable was not significant in the univariate analysis.

Regarding health education service utilization, the main influencing factors include age, education level, household registration, migration scope, income, medical insurance, and region. Key factors contributing to inequality in health education utilization, based on the decomposition results, are higher income (87.4%), employment (68.5%), education (high school and above; 58.0%), and household registration (26.7%), all showing positive concentration coefficients. In terms of health records, the main influencing factors are gender, age, education level, and region; the modified index decomposition shows that the eastern region, employment, and inter-provincial migration are the key factors contributing to inequality, with total contribution rates of 94.4%, 50.2%, and 21% respectively. Medical insurance, higher income, and high school education or above also cause pro-rich inequality in the utilization of health record services, but the contribution rate is small, with total contributions of 1.29%, 0.861%, and 0.977%, respectively. For family doctor contracts, the main influencing factors are migration scope, income, and medical insurance. Based on the decomposition results, higher education and inter-provincial mobility are key contributing factors, with contribution rates of 57.8% and 31.1%, respectively. Regarding inpatient service utilization, the main influencing factors include gender, age, education level, marital status, employment, migration scope, and region. Based on the modified index decomposition, higher education, higher income, and inter-provincial mobility are important factors contributing to inequality in family doctor contract service utilization, with contribution rates of 13.4%, 17.0%, and 40.9%, respectively.

### Health outcomes of basic public health service utilization among migrants

#### Impact of basic public health service utilization on migrants’ health

To assess the health outcomes of public health service utilization among migrants, this study examines the impact of utilizing four basic services (including health education) on their health status and health equity. In this analysis, service utilization serves as the independent variable, while health outcomes are the dependent variable. This study first explores the health differences between different public health service programs, and the results are shown in Table 5. It can be seen that the health differences of the migrant population between the utilization of health education, health records, family doctor contracting, and hospitalization services are all statistically significant.

**Table 5:** Health disparities between different public health service programs.

Variables		Health						χ2	P value
		Unhealthy		Basically healthy		Healthy			
		n	%	n	%	n	%		
Health education	No	131	29.84	180	17.96	411	15.85	50.0107	0.000
	Yes	308	70.16	822	82.04	2182	84.15		
Health record	No	256	58.30	636	63.50	1709	65.90	10.085	0.039
	Yes	183	41.70	366	36.50	884	34.10		
Family doctor contracting	No	340	77.45	845	84.33	2191	84.50	14.0653	0.001
	Yes	99	22.55	157	15.67	402	15.50		
Utilization of inpatient services	No	258	58.77	736	73.45	1784	68.80	30.7082	0.000
	Yes	181	41.23	266	26.55	809	31.20		

Combining the results of the univariate analyses in Tables 2, 5, an ordered logistic regression analysis is conducted using statistically significant variables as independent variables and migrants’ health status as the dependent variable, with results shown in Table 6. Among them, migrant populations who receive health education are more likely to have better health compared to those who do not receive health education (OR = 1.229, 95% CI = 0.028 −0.386); the migrant population with health records are more likely to have better health compared to those without health records (OR = 1.139, 95% CI = −0.021-0.282); and the migrant population with local family doctors are more likely to have better health compared to those who do not have a contract with a local family doctor (OR = 1.131, 95% CI = 0.079-0.466). Additionally, while receiving inpatient services is associated with better health status among migrants, this association is not statistically significant (*P* = 0.604). These analysis results indicate that access to public health services helps improve the health status of migrants.

**Table 6:** Ordered logistic regression results of public health service utilization affecting the health of the Mobile population.

Variables		β	OR	95%CI	P	β	OR	95%CI	P	β	OR	95%CI	P	β	OR	95%CI	P
Unhealthy		2.305	10.024	1.709—2.901	0.000	2.456	11.658	1.824—3.089	0.000	2.505	12.244	1.887—3.123	0.000	2.307	10.044	1.202—2.912	0.000
Basically healthy		4.405	81.859	3.794—5.016	0.000	4.537	93.410	3.889—5.184	0.000	4.605	99.283	3.973—5.238	0.000	4.404	81.778	3.785—5.024	0.000
Health education	No（reference）																
	Yes	0.207	1.229	0.028—0.386	0.023												
Health record	No（reference）																
	Yes					0.130	1.139	−0.021—0.282	0.091								
Family doctor contracting	No（reference）																
	Yes									0.272	1.313	0.079—0.466	0.006				
Utilization of inpatient services	No（reference）																
	Yes													0.042	1.043	−0.115—0.199	0.604
Other Variables		Control															

### Impact of basic public health service utilization on health equity among migrants

**Figure 7:**
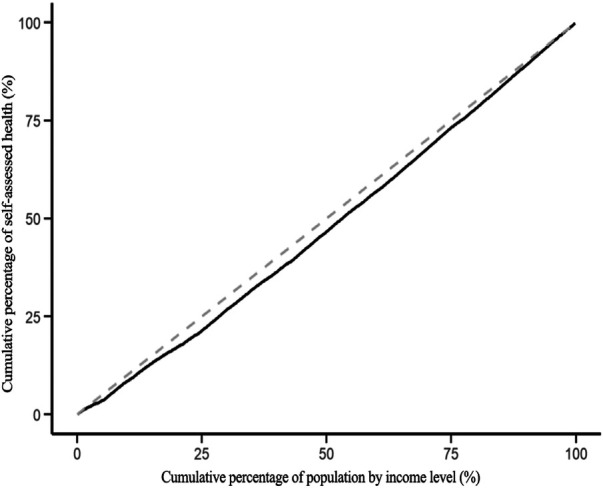
Concentration curve for health equity.

To explore the contribution of public health service utilization of the migrant population to their health inequality, this study first plots the health inequality curve of the migrant population, see Figure 7. The health concentration index of the migrant population is 0.046, as shown in Figure 1. In this study, the health indicator is a positive measure where higher values indicate better health status. The health concentration curve falling below the line of equality indicates that higher-income individuals have better health status, demonstrating pro-rich health inequality. The migrants’ health concentration curve lying below the absolute equality line indicates that health inequality favors those with higher incomes, with healthier migrants being concentrated among higher-income groups.

The decomposition results of the equity of health status of the migrant population are shown in Table 7. The elasticity coefficient and concentration index of health education on health are positive, indicating that health education has a direct positive impact on the health of the migrant population. At the same time health education improves the health of the high-income migrant population to a greater extent. Health education exacerbates health inequality, with a contribution rate of 1.7%. The concentration index of health records and family doctor contracting on health is negative, indicating that these two public health services reduce health inequality among the migrant population, with contribution rates of 1.3% and 1.6% respectively.

**Table 7:** Decomposition of the concentration index for equity in health status of the mobile population.

Variables		Coefficient of elasticity	CI	Degree of contribution	Contribution rate（%）	Coefficient of elasticity	CI	Degree of contribution	Contribution rate（%）	Coefficient of elasticity	CI	Degree of contribution	Contribution rate（%）
Health education	No (reference)												
	Yes	0.058	0.014	0.001	1.700								
Health record	No (reference)												
	Yes					−0.01	−0.06	0.001	1.300				
Family doctor contracting	No (reference)												
	Yes									−0.006	−0.129	0.001	1.600
Other Variables	Control												

### Heterogeneity analysis of the impact of basic public health service utilization on migrants’ health equity

To analyze the heterogeneous impacts of basic public health service utilization on the health equity of the migrant population, this study further explores the four dimensions of urban-rural, age, education, and income. The results are shown in Table 8. From the analysis of the urban-rural heterogeneity results, the contribution rate of health education to health inequality among the urban migrant population is 1%, and the contribution rate to health inequality among the rural migrant population is 1.9%. The contribution of health records and family doctor contracting to health inequality among rural-urban migrants is also greater than that of urban, indicating that all three public health service utilizations expand health inequality among rural migrants to a greater extent.

**Table 8:** Heterogeneity analysis of the impact of basic public health service utilization on health equity.

Variables		Urban/Rural		Age			Education level		Income		
		urban	rural	lower	middle	Higher	High school and lower	University and above	lower	middle	Higher
Health education	Coefficient of elasticity	0.038	0.069	0.002	0.057	0.015	0.082	0.008	0.088	0.032	0.021
	CI	0.007	0.015	−0.002	0.013	0.010	0.019	−0.003	0.018	<0.001	−0.004
	Degree of contribution	<0.001	0.001	<0.001	0.001	<0.001	0.002	<0.001	0.002	<0.001	<0.001
	Contribution rate（%）	1.000	1.900	−0.100	1.900	0.300	3.100	−2.600	0.044	<0.001	−1.100
Health record	Coefficient of elasticity	0.003	−0.021	−0.001	−0.002	0.017	−0.011	0.002	−0.009	−0.400	−0.600
	CI	−0.056	−0.063	−0.073	−0.035	0.023	−0.026	−0.053	−0.027	<0.001	−5.800
	Degree of contribution	<0.001	0.001	<0.001	<0.001	<0.001	<0.001	<0.001	<0.001	<0.001	<0.001
	Contribution rate（%）	−0.500	2.300	0.009	0.200	0.700	0.600	−16.500	0.007	<0.001	4.600
Family doctor contracting	Coefficient of elasticity	0.002	−0.012	<0.001	−0.001	0.010	−0.009	0.003	−0.006	−0.004	0.001
	CI	−0.121	−0.132	−0.139	−0.108	0.004	−0.113	−0.092	−0.025	<0.001	−0.095
	Degree of contribution	<0.001	0.002	<0.001	<0.001	<0.001	0.001	<0.001	<0.001	<0.001	<0.001
	Contribution rate（%）	−0.900	2.900	−0.012	0.400	0.100	2.100	−40.500	0.400	<0.001	−0.110

Analyzing the results of age heterogeneity, health education contributed more to health inequality among middle-aged people, followed by older people, with their contribution rates of 1.9% and 0.3%, respectively. Health records reduce health inequality among people of lower and middle ages; and family doctor contracting also reduces health inequality among people of lower and middle ages and makes a greater contribution (0.4%) to reducing middle-aged people's Health inequalities.

Analyzing the results of the heterogeneity of education level, health education contributes more to the health inequality of the migrant population with lower education level, with a contribution rate of 3.1%. The health records reduce the health inequality between the migrant population with different education levels, and contribute more to the migrant population with lower education level. The family doctor contracting also reduces the health inequality between the migrant population with different education levels, and contributes more to the migrant population with high school and below contributes more (2.1%) to the migrant population.

Analyzing the results from the heterogeneity of income, health education contributes more to health inequality among the low-income population with a contribution rate of 4.4%, but health education reduces health inequality among the high-income migrant population. Health records, on the other hand, reduce health inequality between low-income and high-income migrant populations, but their contribution is greater among high-income populations (0.7% < 4.6%); family doctor contracting services also reduce health inequality between low-income and high-income migrant populations.

## Discussion

This study aims to analyze the equity of migrant populations’ utilization of public health services and their health outcomes, providing support for promoting equitable access to public health services and improving the health of migrant populations. As a developing country with the world's largest migrant population, this empirical evidence from China holds significant practical implications. The findings reveal disparities in migrant populations’ utilization of various public health services. While a higher proportion of migrants receive health education, their access to establishing health records, signing contracts with local family doctors, and utilizing inpatient services remains relatively inadequate. Concurrently, income-related inequalities exist in migrant populations’ utilization of public health services. Specifically, health education services exhibit pro-rich inequality, meaning higher-income migrants utilize these services more frequently. Conversely, health records, family doctor contracts, and inpatient services benefit low-income groups, consistent with existing research (55). This may stem from lower-income migrants deriving greater benefit from public health initiatives like health records and family doctor contracts than their higher-income counterparts. China's public health programs have improved equitable service access for migrants, affirming their right to basic services. However, low uptake of health education among low-income migrants indicates that targeted efforts in this area must be strengthened.

Regarding the health status and equity of migrant populations, this study reveals that migrant health outcomes generally indicate a high level of health, with 89.12% classified as basically healthy or healthy. This underscores the necessity for sustained improvements in migrant health, as health serves as the foundation for better leveraging their human capital advantages (56). However, income-related health inequalities are also significantly present among migrants, with high-income groups demonstrating distinct health advantages—a finding consistent with conclusions from related studies (57). Based on the social determinants of health, health inequalities among migrant populations exhibit broad similarities globally. Studies in countries like India and Brazil indicate that socioeconomic status, educational attainment, and access to health information are key determinants of migrant health (58–60). These studies reveal that low income, low educational attainment, and limited access to health information are primary drivers of health inequalities among migrant populations. Addressing this issue requires improving the accessibility and equity of public health services. Therefore, advancing the health of migrant populations requires a dual focus: improving overall health outcomes while specifically reducing health disparities across income groups within this population.

Regarding health outcomes from public health service utilization, an analysis of the relationship between various public health services and health reveals that all services—whether health education, health records, family doctor contracts, or inpatient service utilization—can contribute to improving the health status of the migrant population. Moreover, preventive services (health education, health records, family doctor contracts) exert a greater impact on migrant health than curative services (inpatient service utilization). This indicates that public health services centered on “preventing disease before it occurs” are more effective in improving migrant health, as preventive services shift disease prevention upstream while significantly reducing disease risk and burden (61). This aligns with the “prevention-first” philosophy advocated by Healthy China. Second, analyzing the impact of public health service utilization on health equity among migrant populations reveals that health education exacerbates health inequalities, with a contribution rate of 1.7%. Conversely, health records and family doctor contracts reduce health inequalities among migrants, with contribution rates of 1.3% and 1.6%, respectively. The above findings indicate that China's policy of equalizing public health services for the migrant population has improved their overall health status. Health records and family doctor contracts have narrowed health disparities among migrants, demonstrating that equalization policies have, to some extent, safeguarded the health rights of this group and contributed to advancing the goal of universal health coverage. Notably, health education may paradoxically exacerbate income-related health inequalities among migrants, despite its overall positive association with health. A possible explanation is that the utilization of health education itself remains pro-rich in this study, meaning that higher-income migrants are more likely to access such services. In addition, the benefits of health education may not depend solely on exposure, but also on individuals’ capacity to understand, retain, and translate health information into sustained health behaviors. Migrants with higher income, better education, and more stable living and working conditions are more likely to benefit from health education because they generally have greater health literacy, stronger access to supportive resources, and more opportunities to act on the information received. By contrast, disadvantaged migrants may face barriers such as lower educational attainment, heavier work burdens, unstable residence, and limited continuity of access to local public health services, all of which may reduce both participation in and effective uptake of health education. As a result, although health education can improve overall health, its benefits may accrue disproportionately to socioeconomically advantaged groups, thereby widening existing health inequalities. This interpretation is also consistent with our heterogeneity analysis, which showed that the inequality-enhancing effect of health education was more pronounced among rural, middle-aged, lower-education, and low-income migrant subgroups. With the introduction of the Healthy China 2030 Plan in 2016, promoting equal access to health services, particularly for the migrant population and low-income groups, has become a central goal of national health policy. The Healthy China policy emphasizes “health for all” and encourages a prevention-oriented health strategy, advocating measures such as health education, health management, and family doctor contracts to reduce health disparities. Although the policy has made some progress in increasing the coverage of basic public health services for the migrant population, particularly in the popularization of health records and family doctor contracts, research findings indicate that health education services have failed to effectively reduce health inequalities among low-income groups and have instead exacerbated income disparities. This indicates that while the Healthy China policy has contributed to expanding health education access for the migrant population, significant disparities persist in health education coverage among low-income migrants, particularly regarding access to health knowledge and awareness enhancement. Low-income groups face challenges in fully participating in health education activities due to factors such as low educational attainment, high work stress, and unstable living conditions. This has partially undermined the effectiveness of health education policies. Therefore, to further refine the system for equalizing basic public health services for the migrant population and better leverage its role in safeguarding migrant health and promoting health equity, efforts should focus on enhancing the role of health education in advancing health equity.

Further heterogeneity analysis reveals that health education contributes more significantly to health inequalities among rural migrant populations, middle-aged individuals, those with lower educational attainment, and low-income groups. Health education has widened health disparities among these populations, further indicating that its implementation has yet to fully reach low-income migrants. This indicates that policy equalization has not fully addressed the persistent gaps in health education provision. Future policies should prioritize the actual needs of migrant populations, particularly in expanding health education accessibility. When delivering preventive health services, greater emphasis must be placed on segmenting population characteristics and implementing targeted interventions to enhance health education participation among low-income groups. Health records significantly reduce health inequalities among rural migrant populations, middle-aged and younger individuals, those with lower education levels, and low-income migrants. The role of family doctor contracts in reducing health inequalities is more pronounced among rural migrants, middle-aged individuals, those with low educational attainment, and low-income migrants. These differentiated analysis results for specific subgroups indicate that the utilization of public health services by migrant populations should be guided by the characteristics of targeted subgroups during policy design and resource allocation. This approach aims to provide precise and diversified public services tailored to different migrant groups, thereby enhancing equity among diverse populations.

## Policy recommendations

Based on the findings above, we propose the following policy implications: First, access to underutilized public health services for migrants should be further improved. This recommendation is directly supported by our finding that, although health education has relatively high coverage, the utilization of health record establishment, family doctor contracting, and inpatient services remains insufficient among migrants. Therefore, future policy efforts should focus on expanding the availability and accessibility of these underutilized services, reducing institutional and practical barriers to service use, and preventing essential public health services from being available in principle but inaccessible in practice. Second, the structure of public health services should be optimized with greater attention to both health improvement and health equity. Our results show that all four types of public health services are associated with better health outcomes, and that preventive services generally have stronger health effects than inpatient care. However, the equity effects of different services are not uniform: health education tends to exacerbate health inequality, whereas health record establishment and family doctor contracting help reduce it. Accordingly, future policy should not only expand service coverage, but also improve the equity orientation of service delivery. In particular, health education programs should be redesigned to better reach disadvantaged migrants, while health record management and family doctor services should be further strengthened as effective equity-promoting interventions. Third, policy design and resource allocation should place greater emphasis on subgroup heterogeneity. The heterogeneity analysis indicates that the effects of public health services on health equity differ across rural, middle-aged, lower-education, and low-income migrant subgroups. In particular, the inequality-enhancing effect of health education is more pronounced among disadvantaged groups, while the inequality-reducing effects of health records and family doctor contracting are also more evident in these populations. These findings suggest that a one-size-fits-all service model may be insufficient. More targeted and differentiated interventions should therefore be developed according to the needs and characteristics of specific migrant subgroups. Finally, innovative service delivery approaches should be explored to improve the effectiveness and inclusiveness of public health interventions. Given the barriers that disadvantaged migrants may face in participating in conventional health education activities, digital platforms and flexible service delivery channels could be used to enhance access to health information, strengthen health literacy, and improve engagement with preventive services. In this way, public health policies can better translate empirical evidence into more precise and equitable interventions for migrant populations.

## Conclusion

This study utilizes data from the 2018 CMDS to analyze equity in migrant populations’ utilization of public health services. It further examines the impact of different public health service programs on the health and health equity of migrant populations, yielding the following key conclusions: First, disparities exist in both utilization rates and equity of access across different public health service programs among the migrant population. Health education services show higher utilization among migrants, with a tendency toward higher-income migrants. Conversely, utilization levels for health records, family doctor contract services, and inpatient services remain low, with these three services exhibiting a tendency toward lower-income migrants. Second, the utilization of basic public health services by the migrant population has improved their overall health status. Health education has widened health inequalities among the migrant population, while health records and family doctor contract services have narrowed health disparities among them. Finally, the impact of public health service utilization on health equity among the migrant population exhibits heterogeneity. Public health service content should be enriched based on the health needs of different groups to narrow health disparities between them.

## Limitations

It is worth noting that this study still has certain limitations. First, the cross-sectional survey data used to decompose the Gini coefficient prevents causal analysis of the findings. Future research should incorporate longitudinal survey data to examine long-term trends in migrant populations’ utilization of public health services and health status. Additionally, future research should focus on the actual impact of the Healthy China policy on health inequalities across different income groups, particularly through longitudinal tracking analysis to assess changes in health inequalities before and after policy implementation, thereby providing empirical evidence for policymakers. Second, this study employs self-rated health indicators to reflect individual health status, which inherently carries subjective elements. Future research may incorporate more objective health measurement indicators into the analysis. Third, due to data limitations, outpatient service utilization among migrant populations is excluded from this analysis. As data become more comprehensive, future studies can further examine outpatient service utilization and its equity among migrant populations, exploring the relationship between outpatient services and health outcomes. Fourth, given the complexity of factors influencing public health service utilization and health outcomes, this study incorporates variables supported by data availability and existing research that may affect migrant populations’ public health service utilization and health. Other potential factors may have been omitted and should be considered for future analyses. Fifth, this study does not employ a control group, relying solely on data from the CM DS. While this approach reveals health inequalities in public health service utilization among the migrant population, it may have limitations due to the lack of comparison with resident populations or other groups. For instance, we cannot definitively determine differences in health service utilization between migrants and residents. Future research should incorporate a control group for comparative analysis to better assess health inequalities within the migrant population.

## Data Availability

Publicly available datasets were analyzed in this study. This data can be found here: Data supporting the results of this study came from the National Mobile Population Dynamics Monitoring Survey (CMDS). The datasets generated and/or analyzed during the current study are available in the official website (https://www.ncmi.cn/project/project-showProjectList.html?type=data&amp;id=d3).
